# ICTV Virus Taxonomy Profile: *Plasmaviridae*

**DOI:** 10.1099/jgv.0.001060

**Published:** 2018-04-03

**Authors:** Mart Krupovic, ICTV Report Consortium

**Affiliations:** Department of Microbiology, Institut Pasteur, 25, rue du Dr. Roux, 75015 Paris, France

**Keywords:** *Plasmaviridae*, ICTV Report, taxonomy, Acholeplasma virus L2

## Abstract

The family *Plasmaviridae* includes bacterial viruses with slightly pleomorphic, enveloped virions with a diameter of 50–125 nm. Virions contain infectious, circular, supercoiled dsDNA molecule(s) of approximately 12 kbp. Plasmaviruses infect *Acholeplasma* species, wall-less bacteria of the class Mollicutes, and are released by budding through the cell membrane without causing host cell lysis. Although the temperate bacteriophage Acholeplasma virus L2 of *Acholeplasma laidlawii* is currently the only classified plasmavirus, related prophages reside in the genomes of different *Acholeplasma* species, where they are integrated into tRNA genes. This is a summary of the International Committee on Taxonomy of Viruses (ICTV) Report on the taxonomy of the *Plasmaviridae*, which is available at www.ictv.global/report/plasmaviridae.

## Abbreviation

AVL2, Acholeplasma virus L2.

## Virion

**Fig. 1.**
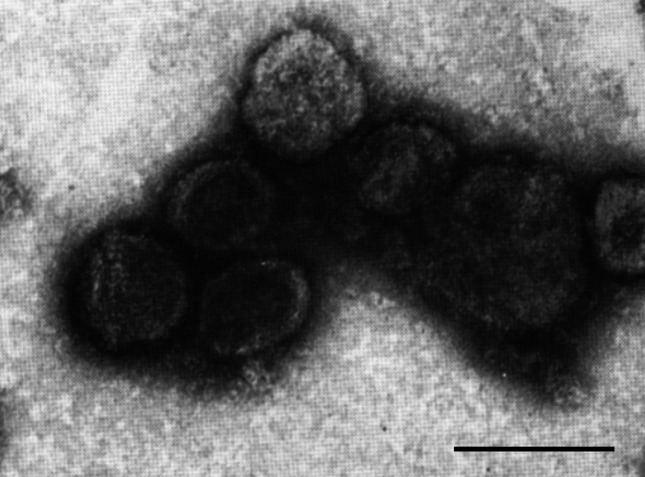
Electron micrograph of Acholeplasma virus L2 virions negatively stained with uranyl acetate. Bar, 100 nm. Modified, with permission from the Microbiology Society, from Gourlay [8].

Virions are quasi-spherical, slightly pleomorphic, enveloped and about 80 nm (range 50–125 nm) in diameter (Table 1, Fig. 1). At least three distinct virion forms are produced during infection, which have the same protein composition, but vary with respect to the number of encapsidated genome copies (from one to three) [1]. Thin sections show virions with electron-dense cores, presumably containing condensed DNA [2]. Virion assembly is coupled to virion release from the infected cells [3]. The absence of a regular capsid structure suggests plasmavirus virions consist of a condensed nucleoprotein bounded by a proteinaceous lipid vesicle.

**Table 1.** Characteristics of the family *Plasmaviridae*

Typical member: Acholeplasma virus L2 (L13696), species *Acholeplasma virus L2*, genus *Plasmavirus*	
Virion	Enveloped, pseudo-spherical and pleomorphic virions (diameter 50–125 nm)
Genome	Circular, supercoiled dsDNA (11 965 bp)
Replication	DNA replication is bidirectional from two origins and is dependent on the host DNA replisome; virions are released by budding
Translation	Translational coupling or re-initiation may be involved in translation of the viral polycistronic mRNAs by the host translation machinery
Host range	*Acholeplasma* species; non-lytic
Taxonomy	Single genus with a single species

## Genome

**Fig. 2.**
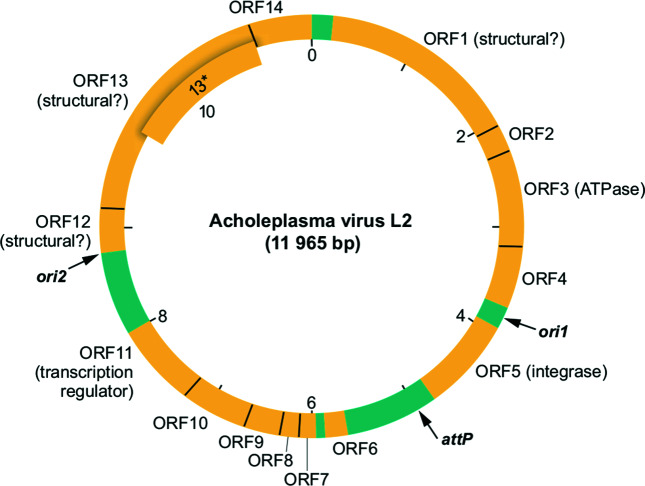
Genome map of Acholeplasma virus L2. The base on the 3′-side of the single *Bst*EII cleavage site is taken as the first base of the DNA sequence. The map also shows locations of the viral attachment site (*attP*) and the two DNA replication origin sites (*ori1* and *ori2*). ORFs are shown in yellow, whereas intergenic regions are in turquoise. ORF13* has a translation start site 295 codons downstream from that of ORF13 and is in the same reading frame.

The genome of Acholeplasma virus L2 (AVL2) consists of a circular, negatively supercoiled dsDNA molecule of 11 965 bp, with a G+C content of 32 %. The genome is infectious when introduced into the cell interior. All 15 annotated ORFs are encoded on one strand and start with an ATG codon (Fig. 2). Each of the ORFs has an upstream Shine–Dalgarno sequence. Several genes are translated from overlapping reading frames. Translational coupling or re-initiation may be involved in translation of the viral polycistronic mRNAs [4].

## Replication

Virus entry is thought to occur by fusion of viral and host cell membranes, resulting in delivery of the nucleoprotein core into the cell [3]. The DNA genome replicates bidirectionally from two *ori* sites, each containing a DnaA box bounded by AT-rich 6-mer repeats [5]. Both *ori* sites are located within intergenic regions of the genome. Replication of the parental DNA is membrane-associated and depends on the host DNA replisome, including DNA polymerase III and DNA gyrase. Plasmaviruses are temperate and establish lysogeny by site-specifically integrating into the host chromosome with the aid of virus-encoded recombinase [6]. Virus production can be reactivated by mitomycin C treatment or UV irradiation. Progeny virions appear to be released by budding through the cell membrane without causing cell lysis [2, 7].

## Taxonomy

The single genus *Plasmavirus* includes the single species *Acholeplasma virus L2*. Related, unclassified, viruses have been reported but their genome sequences are not available. Apparently functional proviruses related to AVL2 are integrated in the genomes of several *Acholeplasma* species. Except for the integrase, plasmaviruses do not share homologous proteins with other known viruses.

## Resources

Full ICTV Online (10th) Report: www.ictv.global/report/plasmaviridae.
